# Comparative Analysis of Visio-Spatial Skills Profiles in Boxing, Karate, and Taekwondo Athletes

**DOI:** 10.3390/jfmk11020190

**Published:** 2026-05-12

**Authors:** Moeketsi Robert Mohlakoana, Gerrit Jan Breukelman, Lourens Millard

**Affiliations:** Department of Human Movement Science, Faculty of Science, Agriculture and Engineering, University of Zululand, KwaDlangezwa 3886, South Africa; breukelmang@unizulu.ac.za

**Keywords:** visio-spatial skills, combat sports, boxing athletes, karate athletes, taekwondo athletes, perceptual-cognitive skills, visual performance

## Abstract

**Background:** Visio-spatial skills (VSS) are essential perceptual-cognitive skills that enable athletes to process visual information, interpret spatial relationships, and execute appropriate motor responses in dynamic sporting environments. In combat sports, athletes must rapidly anticipate and react to an opponent’s actions, making well-developed VSS crucial for optimal performance. Although boxing, karate, and taekwondo share similar competitive characteristics, each discipline presents distinct technical and perceptual demands that may influence the development of specific VSS profiles. This study aimed to investigate whether significant differences exist in VSS profiles among boxing, karate, and taekwondo athletes. **Methods:** A comparative cross-sectional design was used involving 150 amateur combat sport athletes, 50 boxers, 50 karate athletes, and 50 taekwondo athletes. Participants were assessed using a VSS test battery measuring six variables: accommodation facility (AF), saccadic eye movement (SEM), speed of recognition (SR), (HEC), peripheral awareness (PA), and visual memory (VM). Data was analyzed using one-way ANOVA with η^2^, ω^2^, and Cohen’s f effect sizes, and principal component analysis (PCA). **Results:** One-way ANOVA revealed statistically significant differences in five of six VSS (all *p* < 0.001). PA produced the largest sport-specific differentiation (η^2^ = 0.457, Cohen’s f = 0.918), followed by HEC (η^2^ = 0.273, f = 0.612), SR (η^2^ = 0.224, f = 0.537), and SEM (η^2^ = 0.180, f = 0.468). AF yielded a significant moderate effect (η^2^ = 0.108, f = 0.347). VM was the sole non-significant variable (F (2.147) = 0.74, *p* = 0.479, ω^2^ = 0.000), suggesting domain-general encoding processes insensitive to discipline-specific training at this developmental level. Boxing athletes achieved the highest scores in SEM, SR, and PA, while karate athletes led in AF and HEC. PCA revealed a single dominant component (PC1 = 93.91% of variance), confirming that VSS function as a highly integrated perceptual-motor construct rather than independent sub-skills. **Conclusions:** Visio-spatial skills in combat sports are governed by a dominant integrated factor, with discipline-specific variations reflecting unique performance requirements. Visio-spatial skills in combat sport athletes are highly interdependent and largely governed by a single perceptual-motor construct, with discipline-specific profiles observed across boxing, karate, and taekwondo. The findings support the integration of sport-specific, ecologically valid visual training programs targeting key perceptual-cognitive skills, alongside routine assessment to inform athlete development and performance optimization.

## 1. Introduction

Combat sports are characterized by high-speed interactions, rapid decision-making, and continuous adaptation to an opponent’s environment [1]. In such environments, athletes must quickly perceive and interpret visual information to execute appropriate motor responses [2]. Visio-Spatial Intelligence (VSI) is defined as the cognitive capacity to perceive, analyze and mentally manipulate spatial relationships of (objects, people, movement trajectories) [3]. The six VSS which are central to combat sport performance, each address a distinct dimension of visual processing. Accommodative facility defined as the eyes’ ability to rapidly shift focus between near and distant objects to maintain visual clarity, enables combatants to simultaneously monitor opponents, assess distance, and evaluate tactical opportunities during competition [3]. Complementing this, saccadic eye movements, defined as rapid voluntary gaze shifts between fixation points, allow fighters to efficiently scan the visual field, process incoming cues, and maintain situational awareness through fast-paced, unpredictable bouts [4]. Speed of recognition, the capacity to rapidly perceive, identify, and interpret relevant visual information, further distinguishes skilled performers by enabling swift anticipation of opponents’ actions and accurate decision-making under competitive pressure [5]. Hand–eye coordination, which is the integration of visual input with precise motor output of the hands is equally essential, as combatants must synchronize what they see with what they execute to deliver strikes, blocks, and counterattacks with timing and accuracy [4]. Peripheral awareness, defined as the ability to detect and respond to stimuli outside the central visual field while maintaining focus on a primary target, extends this spatial vigilance by allowing fighters to monitor opponents’ positioning and movement without direct fixation, thereby anticipating attacks and identifying defensive openings [5].

Finally, visual memory is defined as the capacity to capture, store, and recall visual information. It supports decision-making in the high-speed, unpredictable context of combat, where the ability to recognize patterns and recall opponent tendencies can determine the outcome of an exchange [6]. Combat sports, though rooted in martial arts philosophy, impose discipline-specific perceptual and motor demands that must directly inform training design [5]. Perceptual anticipation requirements differ substantially across striking, grappling, and weapon-based disciplines, necessitating sport-specific cognitive training adaptations [1]. The ability to detect deceptive movements, such as feints, constitutes a particularly critical requirement in sports involving direct physical confrontation [6]. Perceptual-cognitive expertise must therefore be developed within each discipline’s specific competitive context [2]. Vision training interventions should consequently be tailored to discipline-specific visual demands [4], while multiple object tracking remains a relevant capacity across combat sports requiring simultaneous spatial monitoring of opponents [7].

In combat sports such as boxing, karate, and taekwondo, athletes rely heavily on visual information to anticipate and react to their opponent’s movements [5]. Successful performance requires athletes to detect subtle cues related to body positioning, movement direction, and timing while simultaneously executing defensive or offensive actions [6]. Previous studies in sport science have indicated that athletes participating in fast-paced and open-skill sports often demonstrate enhanced perceptual-cognitive skills compared with non-athletes [7]. These skills are believed to develop through sport-specific training and repeated exposure to complex competitive environments, which demand efficient visual processing and rapid motor responses [8].

Although several studies have investigated visual and perceptual skills in athletes, much of the existing research has focused on individual sports or on general athletic populations [9]. Comparatively fewer studies have examined VSS profiles across different combat sport disciplines [1,9]). This represents an important limitation, as each combat sport places unique perceptual and tactical demands on athletes. For instance, boxing emphasizes rapid hand movements and close-range interactions, karate often requires quick directional changes and precise striking techniques, while taekwondo involves dynamic kicking strategies and greater emphasis on distance management [10]. These differences in technical and tactical demands may influence the development of specific VSS among athletes from different combat sports [11]. Despite the recognized importance of perceptual-cognitive skills in combat sports performance, limited research has directly compared VSS profiles among athletes from boxing, karate, and taekwondo [12,13,14].

Understanding whether differences exist among these groups may provide valuable insights into the perceptual demands of each discipline and contribute to improved athlete training, talent identification, and performance assessment. Identifying sport-specific visio-spatial characteristics may also assist coaches and sport scientists in designing training interventions that better target the perceptual skills required in each combat sport. Therefore, the aim of the present study was to investigate whether significant differences in VSI exist among athletes participating in three combat sport disciplines, namely boxing, karate, and taekwondo.

## 2. Materials and Methods

### 2.1. Study Design

The study followed a cross-sectional observational study which examined differences in VSS between, amateur boxing, karate and taekwondo athletes recruited from the King Cetshwayo Municipal District, KwaZulu Natal, Republic of South Africa. The study involving human participants was reviewed and approved by the Institutional Review Board of the University of Zululand (UZ-REC 0691-008 PGD 2025/12). The participants also provided their written informed consent to participate in this study. The study was conducted in accordance with the principles of the Declaration of Helsinki.

### 2.2. Study Population and Sampling Strategy

The study comprised 150 participants between the ages of 18 and 34 years. These participants were allocated into three groups (n = 50 boxing; n = 50 karate; n = 50 taekwondo), each with a minimum of six months of formal training and were matched according to gender distribution (Table 1). Participants were required to have normal visual acuity or corrected-to-normal vision (20/20 or better) [15]. Exclusion criteria included a history of neurological disorders, uncorrectable visual impairments, prior exposure to (VSS) testing, and engagement in strenuous physical exercise within 48 h preceding the testing sessions. All participants underwent a preliminary optometric screening using Spectrum Eyecare software (version 6.0.0; Digital Optometry, Gqeberha, Republic of South Africa) to assess visual function and confirm eligibility for inclusion. Eligible participants subsequently completed a comprehensive ocular examination to verify the structural health of the visual system, as described previously [16].

### 2.3. Data Collection

**Figure 1:**
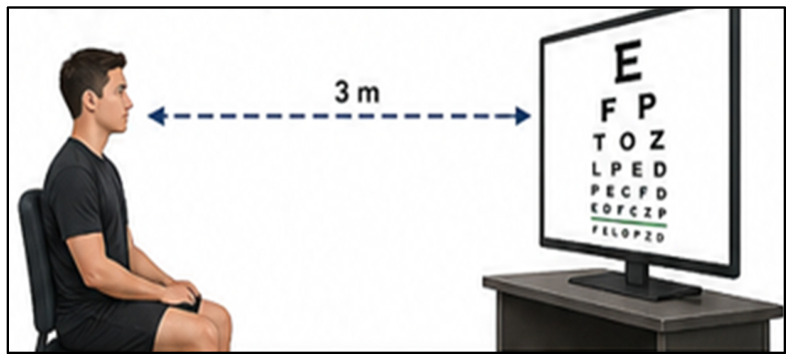
Visual equity test assessment illustration, using the Spectrum Eyecare Software.

Optometric Assessment: Visual acuity was assessed as a measure of the eyes’ ability to resolve fine details, with normal vision defined as 6/6 (m) or 20/20 (ft) [17] (Figure 1). Testing was conducted using Spectrum Eyecare software (Version 6.0.0, Digital Optometry, Gqeberha, Republic of South Africa) calibrated for 3 m viewing distance, with optimized screen size and resolution to ensure standardized testing conditions. Participants were seated 3 m from the display, where rows of optotypes of progressively decreasing size were presented. Beginning with the largest optotypes, participants advanced to smaller sizes until an error criterion was reached. All visual acuity outcomes were reviewed and interpreted by a qualified optometrist to ensure that all participants have a minimum of 20/20 vision.

VSS test battery: Standardized testing procedures were implemented to minimize potential dietary, physical, and psychological confounders. All assessments were scheduled on weekend mornings between 07:00 and 14:00. Participants were tested following a 9 to 12 h overnight fast [3]. Testing was conducted in a quiet, well-lit environment under controlled conditions [18]. Each participant completed two trials for each test, with the highest score retained for analysis. The VSS test battery was designed to comprehensively assess and compare VSS performance between amateur taekwondo athletes and non-athletes, including measures of accommodative facility, saccadic eye movements, speed of recognition, hand–eye coordination, peripheral awareness, and visual memory [3].

**Figure 2:**
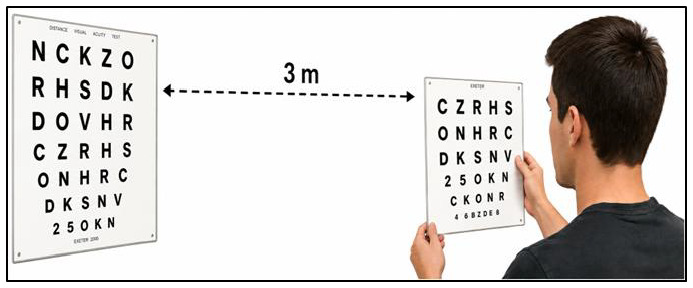
Hart Near-Far Rock Test Chart, an illustration for Accommodation Facility Assessment.

Accommodation facility: Reflects the eyes’ ability to adjust focus between near and distant targets. It was assessed using the Hart Near-Far Rock Test (Figure 2) [17]. A big Hart Chart (Bernell Corp., Mishawaka, IN, USA) was positioned on a wall at eye level, 3 m from the participant, while a second smaller chart was held at arm’s length. Participants read the first letter of each line from the near and far charts alternately for 30 s, with errors recorded. Final scores were calculated by subtracting the number of errors from the total possible score. The test shows acceptable psychometric robustness for applied sport-vision assessment, showing moderate test–retest reliability (r = 0.724), thereby supporting its use in both clinical and athletic performance contexts [19].

**Figure 3:**
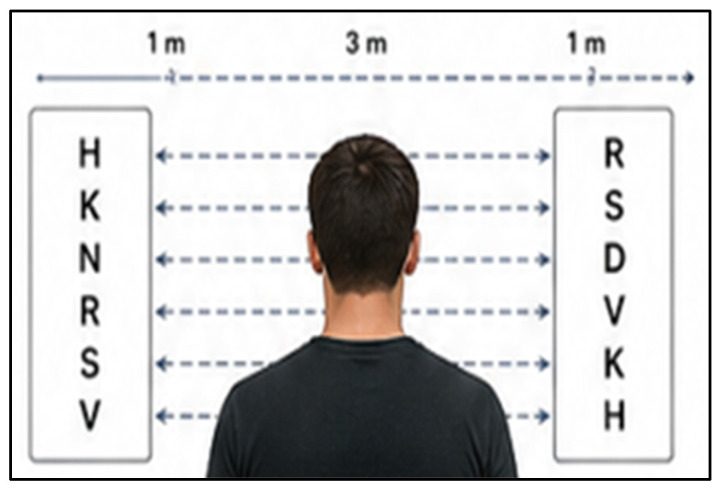
Saccadic eye movement chart, an illustration of the saccadic eye movement assessment.

Saccadic eye movements: Rapid, voluntary shifts in gaze between fixation points were assessed using saccadic eye movement charts [20] (Figure 3). Participants stood 3 m from a wall displaying two charts, each with vertically arranged letters spaced 1 m apart. They verbally identified the first letter on the left chart and then quickly shifted their gaze to the corresponding letter on the right chart, repeating this sequence for 30 s while keeping their head stationary. The number of errors and total letters read were recorded, and final scores were calculated by subtracting errors from the total possible score [3,21]. The test validates moderate test–retest reliability (r = 0.703), supporting its suitability for consistent assessment within sport-vision research and applied performance settings [22].

**Figure 4:**
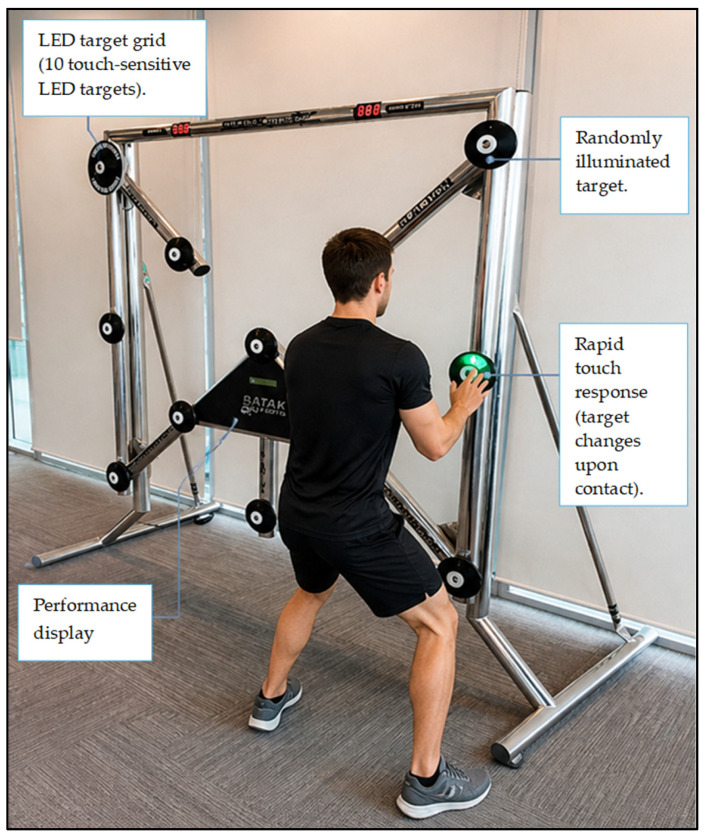
An illustration for speed of recognition, peripheral awareness and visual memory assessment using Batak Pro.

Speed of recognition: The ability to rapidly detect and respond to visual stimuli was evaluated using the Evasion function on the Batak Pro system (Quotronics Limited, Horley, Surrey, UK) [23] (Figure 4). The Batak Pro system is an interactive visuo-motor assessment and training apparatus comprising a wall-mounted panel with multiple touch-sensitive LED targets arranged in a grid; the test has a reliability rating of 0.946 [3]. The system is used to assess and enhance visuo-motor performance, including peripheral awareness, reaction time, hand–eye coordination, and visual scanning, through rapid, goal-directed responses to randomly illuminated visual stimuli under time-constrained conditions [23]. The evasion speed of recognition function was specifically employed to quantify recognition speed. During the speed of recognition assessment, participants stood in front of the Batak Pro panel and were instructed to respond as rapidly as possible to randomly illuminated LED targets. Visual stimuli were presented at unpredictable spatial locations across the grid, requiring rapid visual detection, target recognition, and attentional shifts across central and peripheral vision. Each target changed upon contact, prompting the immediate presentation of a subsequent stimulus. The assessment was performed over a fixed time interval, during which the 100 targets were illuminated sequentially for 1 s [15,24]. The system automatically recorded all response errors, applied real-time score deductions, and generated the final performance score, thereby ensuring objective and standardized data achievement.

Peripheral awareness: The Batak Pro System Accumulator protocol was used to assess peripheral awareness, by requiring participants to detect and respond to randomly illuminated LED targets presented across a broad visual field (Figure 4). Participants stood in front of the panel and were instructed to maintain global visual awareness while rapidly responding to stimuli appearing at unpredictable central and peripheral locations. Each target remained illuminated until contacted, after which a subsequent target was activated at a new spatial position. Participants completed two trials separated by a 5 min recovery interval, during which the system measured the number of correctly executed target contacts within a 60 s period. The Batak Pro System Accumulator protocol automatically records the score. The highest score attained was retained for the final analysis. The test demonstrates high reliability (r = 0.885), indicating strong test–retest consistency and supporting its sturdiness for performance assessment [22].

**Figure 5:**
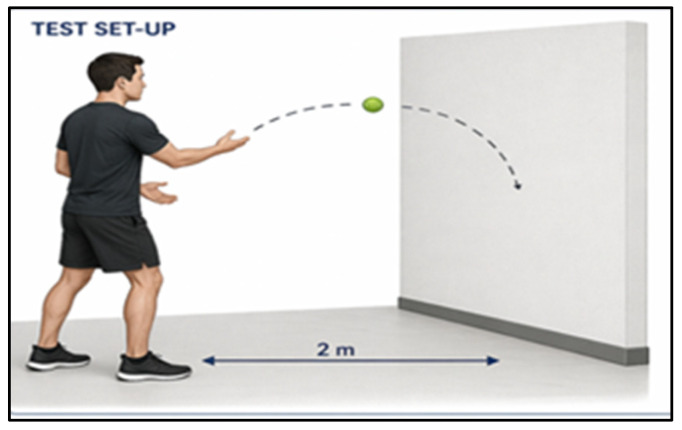
Tennis ball wall test, an illustration for hand–eye coordination assessment.

Hand–eye coordination : Reflects the capacity to integrate visual input with precise and timely motor execution (Figure 5). In this study, it was evaluated using the Tennis Ball Wall Test, a validated field-based measure of visuo-motor synchronization and interceptive control [15]. In this assessment, participants stood 2 m from a wall and executed repeated unilateral throws of a tennis ball, attempting to catch the rebound with the contralateral hand. Both hands were evaluated to determine bilateral accuracy and coordination, with performance recorded over a 30 s interval [3]. Scores ranged from 0 to 60 catches. The test demonstrates moderate reliability (r = 0.708), supporting its use as a consistent measure of hand–eye coordination in applied sport settings.

Visual memory: Defined as the capacity to transiently encode, store, and retrieve visual spatial information. It was assessed using the Flash Memory protocol on the Batak Pro system; the test has a reliability of 0.735 [21] (Figure 4). At the start of each trial, participants adopted a standardized ready position in front of the Batak Pro unit. A double-beep auditory cue signaled the pending onset of the visual stimulus, preparing the participant for stimulus presentation. Immediately following the cue, six randomly distributed targets on the Batak Pro panel were simultaneously illuminated for the duration of 0.5 s. This brief presentation phase required rapid visual scanning, spatial encoding, and attentional focus, as no responses were permitted while the targets were illuminated. Once the illumination period ended, all targets were extinguished, marking the beginning of the recall and response phase [24]. During this phase, participants were required to retrieve previously encoded visio-spatial information from short-term memory and manually activate the prior illuminated targets in any sequence. Responses were completed across five response frames, with performance contingent entirely on accurate spatial recall in the absence of concurrent visual cues. The Batak Pro system automatically scores. Two trials were performed with a 5 min recovery interval, and the highest score achieved was retained for final analysis.

### 2.4. Data Analysis

All statistical analyses were conducted using IBM SPSS Statistics (Version 31.0.1; IBM Corp., Armonk, NY, USA) and XLSTAT (Addinsoft, Paris, France). A one-way analysis of variance (ANOVA) was conducted to examine differences in visio-spatial skill (VSS) performance across three combat sport disciplines (boxing, karate, and taekwondo; n = 50 per group; N = 150), with sport discipline as the independent variable and each VSS domain as a dependent variable. Effect sizes were quantified using eta-squared (η^2^) and bias-corrected omega-squared (ω^2^), with Cohen’s f computed to facilitate interpretation against established benchmarks (small: f = 0.10; medium: f = 0.25; large: f ≥ 0.40; Cohen, 1988 [25]). Statistical significance was set at α = 0.05, and post hoc pairwise comparisons were conducted using the Tukey Honestly Significant Difference (HSD) procedure to control familywise error rate.

Associations among visio-spatial skills (VSS), including visual memory, saccadic eye movement, accommodation facility, peripheral awareness, speed of recognition, and hand–eye coordination were examined using the Pearson correlation coefficient. To further examine the multidimensional structure of the dataset, Principal Component Analysis (PCA) was performed as a data reduction and pattern recognition technique. The PCA generated eigenvalues, component loadings, and the percentage of variance were explained for each principal component. Components with the highest eigenvalues were considered the most influential in explaining variability within the visio-spatial performance measures. The contribution of each variable to the principal components was interpreted based on their loading coefficients. To visualize the multivariate relationships among skills and combat sport groups, a PCA biplot was constructed. The biplot simultaneously displayed the distribution of athletes (or sport categories) and the direction and magnitude of the VSI within the same graphical space. This graphical representation enabled the identification of clusters, associations, and contrasts among the combat sports in relation to their visio-spatial performance profiles.

## 3. Results

The demographic characteristics of the participants was composed of three combat sports (boxing, karate, and taekwondo) (Table 1). Each group consisted of 50 participants with an identical gender distribution of 35 males (70%) and 15 females (30%). This balanced distribution across groups ensured comparability in terms of gender representation during subsequent analyses. The mean age of the participants ranged between 20.2 ± 1.61 and 22.93 ± 2.34 years across the groups. Among non-athletes, males had a mean age of 21.65 ± 2.43 years while females averaged 22.93 ± 2.34 years. In the boxing group, the mean age was 22.14 ± 3.14 years for males and 21.53 ± 2.38 years for females. Karate athletes showed similar age characteristics, with males averaging 22.02 ± 2.77 years and females 22.06 ± 2.31 years. The taekwondo group showed a slightly younger female cohort (20.2 ± 1.61 years) compared with the other groups, while male taekwondo athletes had a mean age of 22.08 ± 2.39 years. Generally, the age distribution across all four groups was relatively homogeneous, with mean ages clustered around early adulthood (approximately 20–23 years). The comparable age ranges suggest that potential age-related confounding effects on visio-spatial performance measures were minimized.

The results indicate variations in visio-spatial performance across the three combat sports: boxing, karate and taekwondo (Table 2). Boxing athletes led in saccadic eye movement (53.32 ± 3.57), speed of recognition (62.44 ± 5.86), and peripheral awareness (68.78 ± 5.75), while karate athletes recorded the highest scores in accommodation facility (42.18 ± 3.88) and hand–eye coordination (43.18 ± 4.15). Taekwondo athletes returned the lowest means across most variables. Visual memory was the sole exception, with scores that were practically equivalent across disciplines (boxing: 53.64 ± 5.26; karate: 53.22 ± 6.78; taekwondo: 52.16 ± 6.64). Between-group differences were statistically significant for five of the six VSS variables, with effect sizes ranging from moderate to very large. Peripheral awareness produced the greatest sport-specific differentiation (η^2^ = 0.457, ω^2^ = 0.448, Cohen’s f = 0.918), accounting for 45.7% of total between-group variance. Large effects were observed for hand–eye coordination (η^2^ = 0.273, f = 0.612), speed of recognition (η^2^ = 0.224, f = 0.537), and saccadic eye movement (η^2^ = 0.180, f = 0.468), confirming that visuo-motor integration, perceptual decision-making, and oculomotor control are meaningfully shaped by discipline-specific training demands. Accommodation facility yielded a significant moderate effect (η^2^ = 0.108, f = 0.347), suggesting that near-far focal adaptation, while training-sensitive, retains greater cross-disciplinary commonality than the preceding skills. Visual memory was the sole non-significant variable (F (2.147) = 0.74, *p* = 0.479, ω^2^ = 0.000), indicating that short-term visio-spatial encoding is essentially equivalent across disciplines and may be governed by domain-general cognitive mechanisms that are insensitive to sport-specific training at this level of athletic development.

### 3.1. Correlation, Principal Component and Biplot Analysis

#### Correlation Among Visio-Spatial Skills

The correlation analysis is one of the striking approaches that can yield adequate evidence on the associations among the VSS measured across boxing, karate, and taekwondo athletes (Table 3). The results demonstrate very strong positive correlations among most of the visual performance skills, indicating that these perceptual abilities are highly interrelated.

Extremely high correlations were observed between visual memory (VM) and speed of recognition (SR) (r = 0.998), as well as between saccadic eye movement (SEM) and speed of recognition (r = 0.998). Similarly, visual memory and accommodation facility (AF) showed a very strong relationship (r = 0.996), suggesting that athletes with better visual memory also tend to display superior accommodative flexibility. Strong correlations were also observed between hand–eye coordination (HEC) and several visual skills, including visual memory (r = 0.993) and accommodation facility (r = 0.996). This indicates that improved visual processing ability is closely associated with more effective visuo-motor coordination. Although slightly lower than the other skills, peripheral awareness (PA) still demonstrated substantial correlations with the remaining visual skills. For example, PA correlated with saccadic eye movement (r = 0.817), hand–eye coordination (r = 0.806), and speed of recognition (r = 0.789). These values that remain above the threshold (r ≥ 0.6) are considered significant in this study. The correlation matrix indicates that all VSI are positively and significantly related, with particularly strong associations among visual processing speed, ocular motor control, and visuo-motor coordination.

### 3.2. Principal Component Analysis

The principal component analysis (PCA) revealed three principal components (PCs) explaining the total variance of the VSS measured in the athletes (Table 4). The first principal component (PC1) showed very high positive loadings for all six skills: Visual Memory (VM = 0.993), Saccadic Eye Movement (SEM = 0.995), Accommodation Facility (AF = 0.986), Peripheral Awareness (PA = 0.843), Speed of Recognition (SR = 0.994), and Hand–Eye Coordination (HEC = 0.994). PC1 had an eigenvalue of 5.635 and accounted for 93.91% of the total variance, indicating that most of the variability among the VSS was captured by this single component.

The second principal component (PC2) showed moderate positive loading for Peripheral Awareness (PA = 0.538) and small negative loadings for the remaining skills (VM = −0.119; SEM = −0.037; AF = −0.151; SR = −0.089; HEC = −0.061). PC2 had an eigenvalue of 0.340 and explained 5.662% of the variance, contributing minimally to the overall structure of the data. The third principal component (PC3) demonstrated very small loadings across all skills (VM = 0.011; SEM = 0.097; AF = −0.068; PA = −0.010; SR = 0.057; HEC = −0.090). With an eigenvalue of 0.025, PC3 explained only 0.424% of the variance.

The cumulative variance explained by the first two components reached 99.576%, while all three components together explained 100% of the variance. However, the overwhelming dominance of PC1 indicates that the VSI are highly interrelated and largely represent a single underlying performance construct.

### 3.3. Biplot Analysis

**Figure 6:**
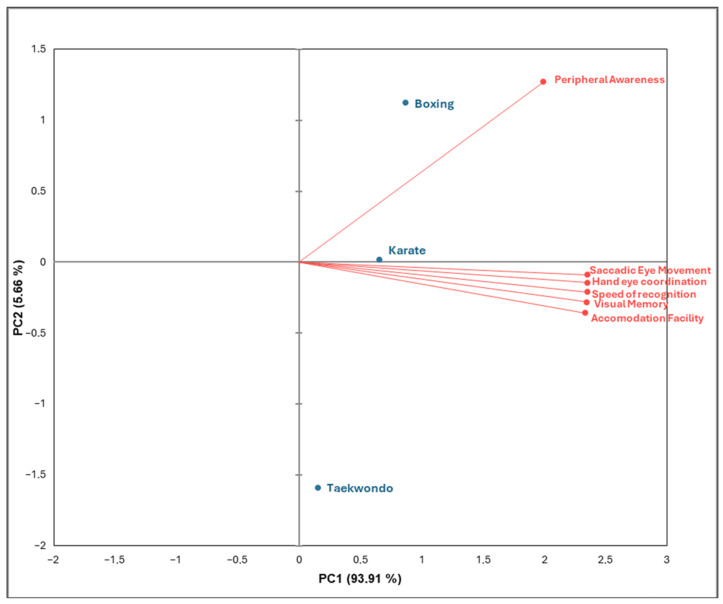
Biplot of boxing, karate, taekwondo and visio-spatial skills.

The principal component analysis (PCA) biplot illustrates the multivariate relationships between selected VSS and three combat sport groups: boxing, karate, and taekwondo athletes (Figure 6). The first principal component (PC1) explains 93.91% of the total variance, while the second principal component (PC2) accounts for 5.66%, indicating that most of the variation in visio-spatial performance among the athletes is captured along PC1.

Most VSS–saccadic eye movement, hand–eye coordination, speed of recognition, visual memory, and accommodation facility, cluster strongly along the positive side of PC1. This clustering suggests that these visual abilities are closely related and tend to co-vary across the athletes. Karate athletes are positioned closest to the origin but slightly toward the positive direction of PC1, indicating a moderate association with these visual performance skills.

Boxing athletes appear separated from the other groups along PC2 in the positive direction and align closely with peripheral awareness, which loads strongly in that direction. This positioning indicates that boxers demonstrate relatively stronger peripheral awareness compared with the other groups. In contrast, taekwondo athletes are in the negative direction of PC2 and relatively distant from the cluster of VSS. This positioning suggests comparatively lower association with the measured visual skills in the present dataset.

The PCA indicates distinct sport-specific visual profiles, with boxers strongly associated with peripheral awareness, karate athletes showing moderate association with multiple VSS, and taekwondo athletes showing weaker associations with the assessed visual performance skills.

## 4. Discussion

The current study examined differences in visio-spatial skills (VSS) among athletes participating in boxing, karate, and taekwondo to identify discipline-specific profiles. The findings revealed clear variations across the three combat sports, indicating that distinct technical, tactical, and perceptual demands may differentially shape VSS. These results contribute to the limited body of literature on the role of VSS in athletic performance, particularly within dynamic, high-speed sporting contexts. This discussion contextualizes the observed differences within existing literature and considers their implications for athlete development, performance optimization, and sport-specific training.

A comparable demographic profile across groups reduces the influence of confounding variables. Participants demonstrated a relatively homogeneous age distribution, with mean ages in the early twenties (Table 1), corresponding to peak physical performance and optimal perceptual-cognitive functioning [26]. The similarity in age suggests comparable developmental stages, which are important as visio-spatial processing, reaction time, and visual tracking mature during late adolescence and early adulthood [27]. Athletes aged 18–25 typically demonstrated optimal neuromuscular coordination and perceptual processing speed [28]. The slightly younger mean age among female taekwondo athletes (20.2 ± 1.61 years) may reflect earlier competitive progression within this discipline [29]. This uniformity strengthens internal validity, ensuring that differences in VSS are more likely attributable to training adaptations rather than age-related factors [30].

Gender distribution was consistent across groups (70% male, 30% female), ensuring methodological balance and minimizing gender-related bias [31]. This distribution reflects broader participation trends in combat sports, where male athletes predominate [32,33,34,35]. However, increasing female representation remains important for understanding gender-specific adaptations [36].

Following the demographic context, VSS comparisons across disciplines are considered. Accommodation facility, defined as the ability to rapidly adjust focus between distances [37], was highest in karate (42.18 ± 3.88), followed by boxing (41.40 ± 2.04) and taekwondo (39.74 ± 2.68). The slightly higher values in karate and boxing likely reflect greater demands for rapid focal adjustments in close-range interactions [38,39,40]. Frequent shifts in focus between an opponent’s limbs and body may enhance accommodative flexibility through sport-specific adaptations [41]. Lower values in taekwondo may relate to longer engagement distances, where broader visual focus is maintained [3,42]. Nevertheless, accommodative efficiency remains essential for tracking high-speed movements and supporting anticipatory performance [1,9,43,44,45].

Saccadic eye movements, which enable rapid gaze shifts between visual targets [46], were highest in boxing (53.32 ± 3.57), followed by karate (49.86 ± 4.10) and taekwondo (48.06 ± 6.08). The superior performance in boxers reflects the need for continuous monitoring of multiple cues during bouts [38,47]. Efficient gaze strategies allow athletes to prioritize relevant information and enhance anticipation [40,44]. Karate athletes also demonstrated strong saccadic performance, consistent with their reliance on rapid decision-making during sparring [39,48]. Lower values in taekwondo may again reflect greater interpersonal distances and broader gaze strategies [3,42]. Efficient saccadic control supports rapid information extraction and response accuracy [9,45,46,49,50].

Hand–eye coordination, essential for synchronizing visual input with motor actions [51], was highest in karate (43.18 ± 4.15), followed by boxing (42.36 ± 3.91) and taekwondo (37.36 ± 4.63). Higher scores in karate and boxing are likely to reflect the emphasis on hand-based techniques [38,52]. These sports require precise timing, accuracy, and integration of visual cues with upper-limb movements [47,51]. Lower values in taekwondo may be attributed to its reliance on lower-limb techniques [42,53]. Despite this, coordination remains critical for maintaining balance and executing defensive actions [37,54]. Enhanced coordination improves timing and accuracy, influencing performance outcomes [9,55].

Speed of recognition, reflecting rapid interpretation of visual stimuli [56], was highest in boxing (62.44 ± 5.86), followed by karate (57.06 ± 9.89) and taekwondo (52.42 ± 6.76). The fast-paced nature of boxing likely explains these results, as athletes must process and respond to visual cues within very short timeframes [14,38,47]. Recognition speed is closely linked to perceptual anticipation, enabling early detection of movement cues [1,45]. While slightly lower in taekwondo, this ability remains well-developed and supports effective decision-making [3,42].

Peripheral awareness, the ability to detect stimuli outside central vision [23], was the highest in boxing (68.78 ± 5.75), followed by karate (64.56 ± 4.90) and taekwondo (57.20 ± 5.07). The demands of boxing require continuous monitoring of multiple cues, enhancing peripheral processing [47]. This skill is critical for detecting incoming strikes and preventing injury [22,44]. Karate athletes similarly benefit from broad visual field processing [39], while lower values in taekwondo may reflect greater reliance on central vision due to increased distances [3,42]. Nonetheless, peripheral awareness remains important for detecting unexpected movements [45].

Visual memory showed similar values across all groups, suggesting it represents a fundamental perceptual-cognitive ability shared among combat athletes [45]. It supports pattern recognition and anticipation by enabling athletes to recall and interpret opponent behaviors [9,57,58]. This function is essential in dynamic interactions, where athletes continuously adapt to evolving situations [59,60].

Although each visual skill contributes independently, performance depends on their integration within a unified perceptual-cognitive system [9]. Accommodation ensures visual clarity, saccadic movements support scanning, coordination links perception to action, recognition speed facilitates interpretation, peripheral awareness expands visual coverage, and visual memory underpins anticipation [23,46,56,61,62]. Expertise emerges from the efficient interaction of these processes [9,45], with discipline-specific demands shaping their relative development [38,42]. These findings emphasize the importance of incorporating targeted visual training into combat sport programs [41,63,64,65].

### 4.1. Correlation, Principal Component, and Biplot Analysis

#### 4.1.1. Correlation Among Visio-Spatial Skills

Correlation and multivariate analyses (Table 3) indicate strong positive relationships among VSS, supporting their interconnected nature. Visual memory, saccadic eye movement, accommodation facility, speed of recognition, and hand–eye coordination showed particularly strong associations. Near-perfect correlations between visual memory and recognition speed (r = 0.998), and between saccadic movements and recognition speed (r = 0.998), suggest that rapid stimulus recognition is closely linked to efficient visual scanning and information retention [1]. These relationships reflect the importance of pattern recognition and anticipatory processing in combat sports [5].

Strong associations between saccadic movements and recognition speed highlight the role of efficient gaze behavior in enhancing perceptual processing. Similarly, the relationship between visual memory and accommodation facility (r = 0.996) suggests that clear visual input supports effective encoding and retention of information. Hand–eye coordination also showed strong correlations with visual memory (r = 0.993) and accommodation facility (r = 0.996), reinforcing the dependence of motor execution on visual processing [22].

Peripheral awareness demonstrated slightly lower but still significant correlations (r = 0.789–0.817), indicating its supportive role within the broader perceptual system. These findings confirm that VSS operates as an integrated network rather than isolated abilities.

#### 4.1.2. Principal Component Analysis and Biplot

Principal component analysis revealed that PC1 accounted for 93.91% of total variance, indicating a dominant dimension representing overall visio-spatial performance, while PC2 explained 5.66% (Table 4 and Figure 6). Most visual skills clustered along PC1, supporting their interdependence and collective contribution to performance.

Karate athletes were positioned near the origin but slightly toward positive PC1, indicating balanced development across VSS. This reflects the sport’s emphasis on rapid perception, anticipation, and coordinated responses [1]. Boxing athletes were separated along PC2 and aligned with peripheral awareness, emphasizing the importance of monitoring multiple cues in close-range interactions. Taekwondo athletes were positioned negatively along PC2 and further from the cluster, suggesting a comparatively weaker association with the measured VSS, possibly due to the sport’s emphasis on lower-limb techniques and spatial distancing [53].

These findings demonstrate sport-specific perceptual profiles shaped by technical and tactical demands. They also support evidence that perceptual-cognitive skills are adaptable and can be enhanced through targeted training [28]). Visio-spatial skills in combat sports are governed by a dominant integrated factor, with discipline-specific variations reflecting unique performance requirements.

## 5. Conclusions

This study revealed discipline-specific visio-spatial profiles among boxing, karate, and taekwondo athletes, reflecting the distinct perceptual and tactical demands of each sport. These differences highlight the importance of adopting sport-specific approaches when evaluating and training perceptual-cognitive abilities. The study further demonstrates that visio-spatial skills (VSS) in combat sport athletes are highly interdependent, supporting the concept that perceptual processing, ocular motor control, and visuo-motor coordination function as an integrated system underlying performance. Principal component analysis indicated that these skills are largely governed by a single dominant perceptual-motor construct, emphasizing the central role of visual processing in combat sport environments and providing a practical framework for performance assessment and monitoring.

From an applied perspective, the results support the inclusion of structured visual training within athlete development programs. Coaches and practitioners should incorporate targeted and ecologically valid training interventions aimed at enhancing accommodative function, saccadic eye movements, peripheral awareness, recognition speed, and visuo-motor coordination. Given the strong interrelationships among these skills, integrated training approaches that simulate sport-specific perceptual demands are recommended. Additionally, routine assessment of VSS may assist in talent identification, individualized training prescription, and performance optimization. Future research should examine the longitudinal effects of visual training interventions and explore potential variations across age, gender, and competitive level to further inform evidence-based practice in combat sports.

## Figures and Tables

**Table 1:** Demographic characteristics of boxing, karate and taekwondo athletes.

Parameter	Boxing Athletes (n = 50)		Karate Athletes (n = 50)		Taekwondo Athletes (n = 50)	
	Male	Female	Male	Female	Male	Female
Gender, n (%)	35 (70%)	15 (30%)	35 (70%)	15 (30%)	35 (70%)	15 (30%)
Age (y)	22.14 ± 3.14	21.53 ± 2.38	22.02 ± 2.77	22.06 ± 2.31	22.08 ± 2.39	20.2 ± 1.61
Body Mass (kg)	69.54 ± 5.14	70.03 ± 5.82	69.92 ± 6.95	67.30 ± 5.77	68.06 ± 4.33	70.21 ± 3.84
Height (m)	1.73 ± 0.05	1.73 ± 0.05	1.76 ± 0.06	2.34 ± 0.72	1.75 ± 0.04	1.78 ± 0.48
BMI (kg/m^2^)	23.21 ± 1.06	23.41 ± 0.95	22.55 ± 0.69	22.52 ± 0.96	22.24 ± 0.69	22.13 ± 0.49
Experience (y)	2.48 ± 1.06	2.20 ± 1.01	2.26 ± 0.88	2.34 ± 0.72	2.19 ± 0.74	2.13 ± 0.51

y, years; SD, standard deviation; n, number of participants; kg, kilogram; %, percent; m, meters; kg/m^2^, kilograms per square meter; BMI, Body Mass Index; Abbreviations: y, years; SD, standard deviation; n, number of participants.

**Table 2:** Visio-spatial skill comparison in boxing, karate and taekwondo athletes.

VSS	Boxing Athletes (*n* = 50)	Karate Athletes (*n* = 50)	Taekwondo Athletes (*n* = 50)	F	*p*	Cohens’ f	Effect Size
AF	41.40 ± 2.04 ^a,b^	42.18 ± 3.88 ^a^	39.74 ± 2.68 ^b^	8.86	<0.001 ***	0.347	Medium
SEM	53.32 ± 3.57 ^a^	49.86 ± 4.10 ^a,b^	48.06 ± 6.08 ^b^	16.08	<0.001 ***	0.468	Large
HEC	42.36 ± 3.91 ^a^	43.18 ± 4.15 ^a^	37.36 ± 4.63 ^b^	21.19	<0.001 ***	0.537	Large
SR	62.44 ± 5.86 ^a^	57.06 ± 9.89 ^a,b^	52.42 ± 6.76 ^b^	27.56	<0.001 ***	0.612	Large
PA	68.78 ± 5.75 ^a^	64.56 ± 4.90 ^b^	57.20 ± 5.07 ^c^	61.98	<0.001 ***	0.918	Large
VM	53.64 ± 5.26	53.22 ± 6.78	52.16 ± 6.64	0.740	0.479 ns	0.100	Negligible/NS

Data are presented as mean ± SD. Superscript letters (a, b, c) denote homogeneous subgroups from Turkey HSD post hoc tests: groups sharing the same letters do not differ differently (*p* > 0.05). n, number of participants; ns, not significant; VSS, Visio-spatial skills; AF, Accommodation Facility; SEM, Saccadic eye movement; HEC, hand–eye coordination; SR, Speed of recognition; PA, Peripheral awareness; VM, Visual memory. *** *p* < 0.001.

**Table 3:** Correlation coefficient between the visio-spatial skills in boxing, karate and taekwondo.

Variables	VM	SEM	AF	PA	SR	HEC
**VM**	1					
**SEM**	0.993	1				
**AF**	0.996	0.980	1			
**PA**	0.772	0.817	0.751	1		
**SR**	0.998	0.998	0.990	0.789	1	
**HEC**	0.993	0.982	0.996	0.806	0.989	1

Values ≥ 0.6 are deemed to be significantly correlated. VM—Visual Memory; SEM—Saccadic Eye Movement; AF—Accommodation Facility; PA—Peripheral awareness; SR—Speed of recognition; HEC—hand–eye coordination.

**Table 4:** Principal component analysis of visio-spatial skills.

Variables	PC1	PC2	PC3
VM	0.993	−0.119	0.011
SEM	0.995	−0.037	0.097
AF	0.986	−0.151	−0.068
PA	0.843	0.538	−0.010
SR	0.994	−0.089	0.057
HEC	0.994	−0.061	−0.090
Eigenvalue	5.635	0.340	0.025
Variability (%)	93.914	5.662	0.424
Cumulative %	93.914	99.576	100.000

Values ≥ 0.6 are deemed to be significantly correlated. VM—Visual Memory; SEM—Saccadic Eye Movement; AF—Accommodation Facility; PA—Peripheral awareness; SR—Speed of recognition; HEC—hand–eye coordination.

## Data Availability

The data presented in this study are available upon request from the corresponding author to protect the confidentiality of the participants.
